# Validation of the REVEAL Prognostic Models in Systemic Lupus Erythematosus-Associated Pulmonary Arterial Hypertension

**DOI:** 10.3389/fmed.2021.618486

**Published:** 2021-03-04

**Authors:** Jingge Qu, Mengtao Li, Xiaofeng Zeng, Xiao Zhang, Wei Wei, Xiaoxia Zuo, Ping Zhu, Shuang Ye, Wei Zhang, Yi Zheng, Wufang Qi, Yang Li, Zhuoli Zhang, Feng Ding, Jieruo Gu, Yi Liu, Miaojia Zhang, Junyan Qian, Can Huang, Jiuliang Zhao, Qian Wang, Yongtai Liu, Zhuang Tian, Yanhong Wang

**Affiliations:** ^1^Department of Rheumatology and Clinical Immunology, State Key Laboratory of Complex Severe and Rare Diseases, Peking Union Medical College Hospital (PUMCH), Chinese Academy of Medical Sciences and Peking Union Medical College, National Clinical Research Center for Dermatologic and Immunologic Diseases (NCRC-DID), Ministry of Science and Technology, Key Laboratory of Rheumatology and Clinical Immunology, Ministry of Education, Beijing, China; ^2^Department of Rheumatology, Guangdong General Hosptal, Guangzhou, China; ^3^Department of Rheumatology, Tianjin Medical University General Hospital, Tianjin, China; ^4^Department of Rheumatology, Xiangya Hospital, Central South University, Changsha, China; ^5^Department of Clinical Immunology, People's Liberation Army Specialized Research Institute of Rheumatology and Immunology, Xijing Hospital, Fourth Military Medical University, Xi'an, China; ^6^Department of Rheumatology, Ren Ji Hospital, School of Medicine, Shanghai Jiao Tong University, Shanghai, China; ^7^Department of Rheumatology, Ren Ji Hospital, School of Medicine, Shanghai Jiao Tong University, Shanghai, China; ^8^Department of Rheumatology, Beijing Chao-Yang Hospital, Capital Medical University, Beijing, China; ^9^Department of Rheumatology, The First Central Hospital, Tianjin, China; ^10^Department of Rheumatology, The Second Affiliated Hospital of Harbin Medical University, Harbin, China; ^11^Department of Rheumatology and Clinical Immunology, Peking University First Hospital, Beijing, China; ^12^Department of Rheumatology, Qilu Hospital of Shandong University, Jinan, China; ^13^Department of Rheumatology, The Third Affiliated Hospital of Sun Yat-sen University, Guangzhou, China; ^14^Department of Rheumatology and Immunology, West China Hospital, Sichuan University, Chengdu, China; ^15^Department of Rheumatology, The First Affiliated Hospital of Nanjing Medical University, Nanjing, China; ^16^Department of Cardiology, Peking Union Medical College Hospital, Peking Union Medical College and Chinese Academy of Medical Sciences, Beijing, China; ^17^Department of Epidemiology and Bio-Statistics, Institute of Basic Medical Sciences, China Academy of Medical Sciences and Peking Union Medical College, Beijing, China

**Keywords:** systemic lupus erythematosus, pulmonary arterial hypertension, risk stratification, REVEAL model, 1-year survival

## Abstract

No previous studies have investigated the predictive performance of the Registry to Evaluate Early and Long-term Pulmonary Arterial Hypertension Disease Management (REVEAL) prognostic equation and simplified risk score calculator in patients with systemic lupus erythematosus-associated pulmonary arterial hypertension (SLE-PAH). We aimed to validate these prediction tools in an external cohort of patients with SLE-PAH. In this study, the validation cohort consisted of patients with SLE-PAH registered in a prospective, multicenter, nationwide database between November 2006 and May2016. The follow-up of patients was censored at 1 year. Discrimination, calibration, model fit, and risk stratification of the REVEAL prognostic equation and simplified risk score calculator were validated. As a result, a total of 306 patients with SLE-PAH were included. The 1-year overall survival rate was 91.5%. The C-index of the prognostic equation was 0.736, demonstrating reasonably good discrimination, and it was greater than that for the simplified risk score calculator (0.710). The overall calibration slope was 0.83, and the Brier score was 0.079. The risk of renal insufficiency and World Health Organization Functional Class III (WHO FC III) were underestimated, and the risk assigned to a heart rate >92 bpm in the REVEAL prognostic models was not observed in our validation cohort. Both model discrimination and calibration were poor in the very high-risk group. In conclusion, the REVEAL models exhibit good discriminatory ability when predicting 1-year overall survival in patients with SLE-PAH. Findings from both models should be interpreted with caution in cases of renal insufficiency, WHO FC III, and heart rate >92 bpm.

## Introduction

Pulmonary arterial hypertension (PAH) is the most severe complication and one of the leading causes of death among patients with systemic lupus erythematosus (SLE) (1). In Asian countries, SLE-PAH has become the leading cause of connective tissue disease (CTD)-associated PAH due to the high prevalence of SLE in this population (2). Accurate and generalizable risk prediction may contribute to timely identification of patients at risk for poor outcomes, allowing for earlier clinical intervention and potentially improving patient outcomes. However, the clinical characteristics of SLE-PAH differ from those of other forms of PAH. Patients with SLE-PAH exhibit a higher rate of multi-organ involvement, a stronger inflammatory component, and better treatment response to immunosuppressant therapy compared to those with idiopathic PAH (IPAH) (3). Currently, there are no reliable tools for accurately predicting survival in these patients. Therefore, it remains necessary to validate the applicability of PAH survival prediction tools in patients with SLE-PAH.

The Registry to Evaluate Early and Long-term PAH Disease Management (REVEAL) is a multicenter, observational, US-based registry developed to assess the prognosis of patients with PAH (WHO Group 1) (4). Based on the prospectively collected data of 2,716 patients included in the REVEAL database, a prognostic equation and simplified risk score calculator were developed to predict 1-year overall survival (OS) in patients with PAH (5, 6). These two prediction tools, which include the same 19 independent predictors, were determined using multivariable Cox proportional hazards models. Such tools allow for accurate risk prediction at the individual level as well as risk stratification. Detailed estimates of model parameters have also been presented, which allow for validation studies.

In a previous external validation study, the REVEAL model performed well in geographically diverse PAH populations and even in patients with WHO Group 2–5 pulmonary hypertension (7–9). However, in patients with incident systemic sclerosis (SSC)-associated PAH, the model provided good discrimination but poor calibration (10). Moreover, the predictive performance of these tools for SLE-PAH remains unclear. Therefore, the present study aimed to validate the predictive accuracy of the REVEAL model in patients with SLE-PAH.

## Methods

The methods described in this article follow the criteria outlined in the Transparent Reporting of a multivariate prediction model for Individual Prognosis Or Diagnosis (TRIPOD) statement (11).

### Validation Cohort

The Chinese SLE Treatment and Research Group (CSTAR) is a prospective, longitudinal, nationwide register-based study including patients with SLE in 104 centers covering 30 provinces in China (12). Fourteen referral centers participating in the CSTAR were eligible to enroll patients with SLE-PAH (3). Patients with newly diagnosed PAH following right heart catheterization (RHC) who visited the participating centers from November 2006 to May 2016 were enrolled in the CSTAR-PAH cohort. These patients were included in the validation cohort. SLE was diagnosed by a rheumatologist at each CSTAR center, in accordance with the 2012 Systemic Lupus International Collaborating Clinics (SLICC) classification criteria (13). Diagnoses of PAH were based on RHC, as previously described (3). Patients with other forms of pulmonary hypertension identified via a pulmonary function test showing total lung capacity <60% and ventilation perfusion scintigraphy (V/Q) were excluded. We also excluded patients with pulmonary thromboembolism confirmed via computed tomographic pulmonary angiography and those with overlapping CTDs. The researchers at each center guaranteed the integrity and accuracy from their institution, and medical ethics committee approval was obtained according to local regulations.

Baseline was defined as the time of SLE-associated PAH diagnosis confirmed by RHC. At baseline, we obtained information related to the following: demographic characteristics, medical history, physical examination findings, transthoracic echocardiography (TTE) result, pulmonary function test results, hemodynamic measurements from RHC, and serum laboratory results. All patients underwent a comprehensive follow-up clinical assessment at least once a year. The study end-point was chosen based on all causes of death within 1 year after baseline. Causes of death were determined based on clinical records, social security data, and death registries. The follow-up of patients was censored at 1 year after baseline.

### REVEAL Prognostic Model

The REVEAL prognostic equation (6) and simplified risk calculator (5) were assessed in a validation cohort of patients with SLE-PAH in accordance with previously described methods. The 19 predictor variables are shown in Table 1. In the prognostic equation, the 1-year OS of an individual patient's predicted probability was calculated as follows: $P_{1\;year\;suvival}=S_{0\;}{\left(1\right)}^{\exp\left(Z^{\prime }\beta \gamma \right)},\;$ where *S*_0_(1) represents the baseline survivor function (0.9698), γ represents the shrinkage coefficient (0.939), *Z'*β represents the sum of each patient's feature multiplied by β for each of the 19 parameters, and *Z'*βγ is the product of the shrinkage coefficient which represents the prognostic index (PI). Patients were stratified into the following five risk groups according to the predicted probability of individuals: low (≥95%), average (90 to <95%), moderately high (85 to <90%), high (70 to <85%), and very high (<70%) (6). In the risk calculator, the baseline score of each patient was 6, and points are added or subtracted for each predictor variable. Patients were also classified into the following five risk groups: low (score ≤ 7), average (score = 8), moderately high (score = 9), high (score = 10 or 11), and very high (score ≥12) (5).

**Table 1:** Predictors for the REVEAL prognostic equation, simplified risk calculator, and validation cohort.

	**β of Prognostic Equation**	**Points of risk calculator**	**Patients with all causes of death end-points within 1 year (*n* = 26)**	**Patients without all causes of death end-points within 1 year (*n* = 280)**
**WHO group I subgroup**				
FPAH	+0.7737	+2	0	0
APAH-PoPH	+1.2081	+2	0	0
APAH-CTD	+0.4624	+1	26 (100%)	280 (100%)
**Demographics and comorbidities**				
Male >60 y of age	+0.7779	+2	0	0
Renal insufficiency	+0.6422	+1	6 (23%)	10 (4%)
**NYHA/WHO FC**				
FC I	−0.8740	−2	0	14 (5%)
FC III	+0.3454	+1	19 (73%)	129 (46%)
FC IV	+1.1402	+2	3 (12%)	10 (4%)
**Vital signs**				
SBP < 110 mm Hg	+0.5128	+1	10 (38%)	104 (37%)
Heart rate > 92 bpm	+0.3322	+1	6 (23%)	114 (41%)
**6MWD test**				
6MWD > 440 m	−0.5455	−1	2 (8%)	105 (38%)
6MWD<165 m	+0.5210	+1	0	8 (3%)
**BNP**				
BNP < 50 pg/mL or NTproBNP < 300 pg/mL	−0.6922	−2	2 (8%)	57 (20%)
BNP > 180 pg/mL or NTproBNP > 1,500 pg/mL	+0.6791	+1	16 (62%)	109 (39%)
**Echocardiogram**				
Any pericardial effusion	+0.3014	+1	16 (62%)	128 (46%)
**Pulmonary function test**				
Predicted DLCO > 80%	−0.5317	−1	1 (4%)	15 (5%)
Predicted DLCO < 32%	+0.3756	+1	0	6 (2%)
**Right heart catheterization (mm Hg Wood units)**				
mRAP > 20 mmHg	+0.5816	+1	0	3 (1%)
PVR > 32 Wood units	+1.4062	+2	0	0

*REVEAL, Registry to Evaluate Early and Long-term Pulmonary Arterial Hypertension Disease Management; WHO, World Health Organization; FPAH, familial pulmonary arterial hypertension; APAH, associated pulmonary arterial hypertension; PoPH, portopulmonary hypertension; CTD, connective tissue disease; NYHA, New York Heart Association; WHO FC, World Health Organization Functional Class; SBP, systolic blood pressure; 6MWD, 6-minute walk distance; BNP, brain-type natriuretic peptide; NTproBNP, N-terminal-pro hormone BNP; DLCO, diffusing capacity for carbon monoxide; mRAP, mean right atrial pressure*.

It is worth noting that renal function was assessed based on glomerular filtration rate (GFR), which was estimated using the Chronic Kidney Disease-Epidemiology Collaboration (CKD-EPI) equation (14). Renal insufficiency was defined as an eGFR of <60 ml/min/1.73 m^2^. Missing data for any of the 19 predictor variables were assigned to the reference group (coded as “0”), as described in the study related to development of the REVEAL model (6).

### General Statistical Methods

Continuous and categorical variables are expressed as the mean and standard deviation (SD), median and range, or percentages, as appropriate. The follow-up time for each patient was calculated from baseline to the date of reaching the end-point or 1 year after baseline. All statistical analyses were performed using R statistical software, version 3.4.3 (https://www.r-project.org/).

### Validation of the REVEAL Model

The discriminative and predictive abilities of the prognostic equation and simplified risk calculator were assessed based on the C-index (15). A C-index of 0.5 indicates no discrimination, while an index of 1 indicates perfect discrimination. The calibration slope was used to assess the degree of agreement between the observed and predicted hazards of the end-point. Values were estimated by fitting the PI (*Z'*βγ) based on the original prognostic model as a predictor, as follows: ln *h*(*t*) = ln *h*_0_(*t*) + β(*Z'*βγ) (16). A poor calibration slope (β < 1) usually reflects overfitting of the model in the development sample or indicates the contradictoriness of the predictor effects between the development and validation samples. The Brier score evaluates both discrimination and calibration on a scale from 0 to 1, with lower scores indicating better predictive performance (17). The fit of the model was assessed via Cox proportional hazards regression analyses of model covariates, with an offset of the PI, as follows: ln *h*(*t*) = ln *h*_0_(*t*) + *x'*β^*^ + (*Z'*βγ)(16). The coefficient of *Z'*βγ was constrained to 1.

We also validated the ability of each tool for risk stratification. Kaplan–Meier survival curves were constructed to analyze survival differences among the five risk groups.

The five risk groups' hazard ratios were calculated using a Cox proportional hazards regression model with the average risk group as the reference group (16). To further assess calibration in each risk group, the validation cohort was classified into five groups using prognostic equation and the simplified risk score calculator according to the predicted risk as calculated by the prognostic equation (18).

## Results

### Characteristics of the CSTAR-PAH Cohort

In this study, we identified 310 patients with RHC-confirmed SLE-PAH in the CSTAR-PAH cohort. A total of 306 patients from the CSTAR-PAH cohort with confirmed mortality statuses were included in the validation cohort. The baseline characteristics of these patients are shown in Table 2. Among patients with SLE-PAH, 49.4% exhibited World Health Organization Functional Class (WHO FC) grades of III/IV. Patients in the CSTAR-PAH cohort had more patients with newly diagnosed SLE-PAH, were younger, and were more predominantly female than those in the derivation cohort. All patients in the validation cohort were from China and were of Han origin. In addition, patients in the validation cohort were more likely to have a greater 6-minute walk distance (6MWD), lower right atrial pressure, higher brain-type natriuretic peptide (BNP) or N-terminal (NT)-pro hormone BNP (NT-proBNP) levels, and a higher proportion of patients with pericardial effusion than those in the derivation cohort. No patients with SLE-PAH exhibited PVR > 32 Wood units. None of the patients were men >60 years of age, and none had familial PAH or associated PAH-portopulmonary hypertension.

**Table 2:** Baseline clinical characteristics of the validation cohort.

**Clinical characteristics**	**Validation cohort (*n* = 306)**	**Development cohort (*n* = 2,716)**
Newly diagnosed	79 (25.8%)	367 (13.5%)
Female	304 (99.3%)	2,135 (78.6%)
Age, y	35.0 ± 10.1	50 ± 17
**Modified NYHA/WHO FC**		
I	14 (4.6%)	210 (8.5%)
II	132 (43.1%)	936 (37.8%)
III	138 (45.1%)	1,194 (48.2%)
IV	13 (4.3%)	136 (5.5%)
6MWD, m	409 ± 96	370 ± 127
Right atrial pressure, mm Hg	5.9 ± 4.5	8.6 ± 5.3
Mean pulmonary artery pressure, mm Hg	46.9 ± 12.1	49.5 ± 14.8
Pulmonary vascular resistance, Wood units	11.0 ± 5.7	10.5 ± 6.6
Pulmonary capillary wedge pressure, mm Hg	8.1 ± 3.9	9.6 ± 4.0
Serum NTproBNP level, pg/mL	1,848 ± 2,639	1,455 ± 3,296
Serum BNP level, pg/ml	588 ± 1,343	286 ± 530
Carbon monoxide diffusing capacity, % predicted	58.7 ± 16.2	59.9 ± 23.4
SBP, mm Hg	113.7 ± 17	116 ± 17
Heart rate, beats/min	90.9 ± 13.6	83 ± 15
Pericardial effusion, yes	143 (46.7%)	532 (25.3%)
Renal insufficiency	16 (5.2%)	109 (4.0%)

*NYHA, New York Heart Association; WHO FC, World Health Organization Functional Class; 6MWD, 6-minute walk distance; NTproBNP, N-terminal-pro hormone BNP; BNP, brain-type natriuretic peptide; SBP, systolic blood pressure*.

### Survival

A total of 26 patients reached the end-point, and the 1-year OS rate was 91.5%. Four patients did not undergo follow-up evaluation after baseline. The probability of 1-year OS was similar to that reported for patients with PAH in REVEAL (91.0%).

### Estimates of Model Validation

The C-index for the prognostic equation in the validation cohort was 0.736, indicating similar discriminatory ability when compared with that for the derivation cohort (0.744). For the risk calculator, the C-index in the validation cohort was 0.710, which was lower than that in the derivation cohort (0.735). The calibration slope of the prognostic equation was 0.83, suggesting that the predictions were too extreme (19). The Brier score for the prognostic equation was 0.079, indicating fair performance.

The model with PI offset shows that a joint test including all predictors resulted in a chi-square value of 25.2 and *P-*value of 0.03, indicative of a marginal model fit. The *P*-value for renal insufficiency was <0.0001 (β^*^ = 2.4270), that for New York Heart Association (NYHA)/WHO FC III was 0.0339 (β^*^ = 1.1566), and that for heart rate >92 bpm was 0.0140 (β^*^ = −1.2242). These were the only three covariables exhibiting statistical significance, indicating poor model fit for these three predictors in the validation cohort. The positive β^*^ of the two covariables indicates that patients with renal insufficiency and NYHA/WHO FC III had poorer survival in the validation cohort than in the derivation cohort. In contrast, the negative β^*^ of the covariable means that a heart rate >92 bpm was not as related to poorer outcome in the prognostic model in CSTAR-PAH cohort as in the derivation cohort.

**Figure 1:**
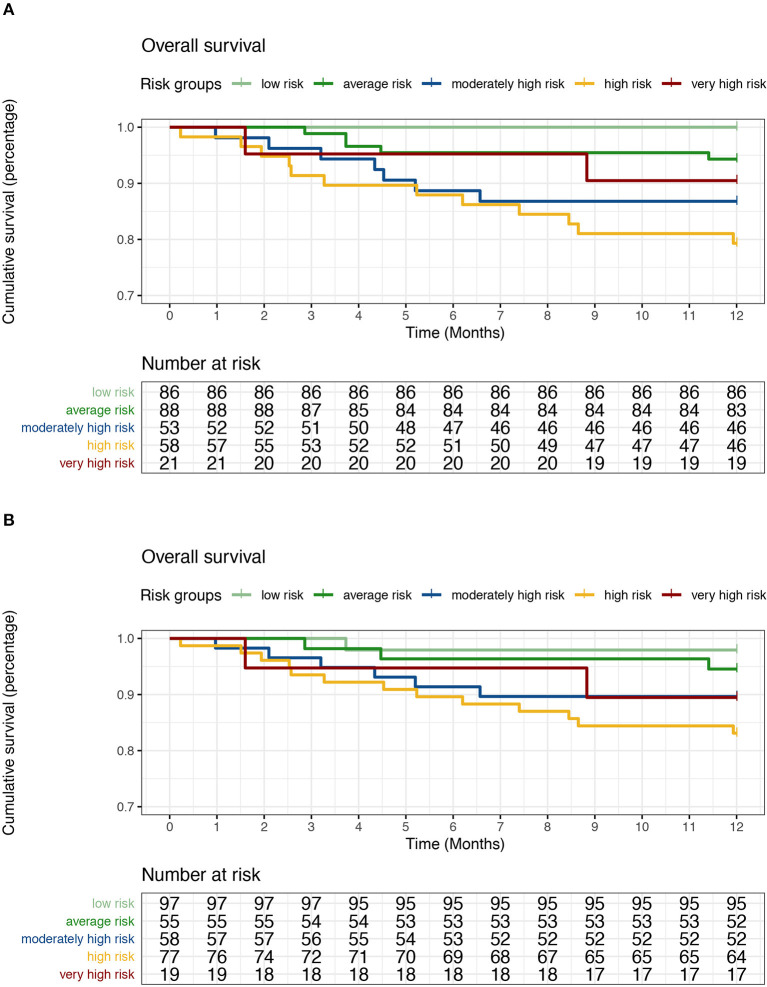
Survival by risk group according to the prognostic equation **(A)** and simplified risk calculator **(B)**.

Further statistical analysis revealed the applicability of risk stratification in the validation cohort. The Kaplan–Meier survival curves of the five groups classified by the REVEAL prognostic equation and simplified risk score calculator are shown in Figure 1. In both curves, low risk, average risk, moderately high risk, and high risk aided in identifying patients with poorer survival in the validation cohort. The survival of the very high-risk group in the validation cohort was not as low as that in the derivation cohort. Instead, the survival rate of the very high-risk group was between that for the average and moderately high-risk groups. Hazard ratios for the probability of 1-year OS across the five groups (Table 3) indicated that the high-risk group exhibited the worst survival. Therefore, although the REVEAL prognostic equation has good overall discriminative ability, discrimination for patients in the very high-risk group was not accurate in the prognostic equation or risk calculator.

**Table 3:** Hazard ratios for each risk group.

	**Hazard ratio (95% CI) of prognostic equation**	**Hazard ratio (95% CI) of risk calculator**
Low risk	–	0.37 (0.06–2.24)
Average risk	Referent	Referent
Moderately high risk	2.44 (0.78–7.70)	1.97 (0.49–7.89)
High risk	3.95 (1.39–11.20)	3.29 (0.94–11.57)
Very high risk	1.71 (0.33–8.82)	1.98 (0.33–11.90)

**Figure 2:**
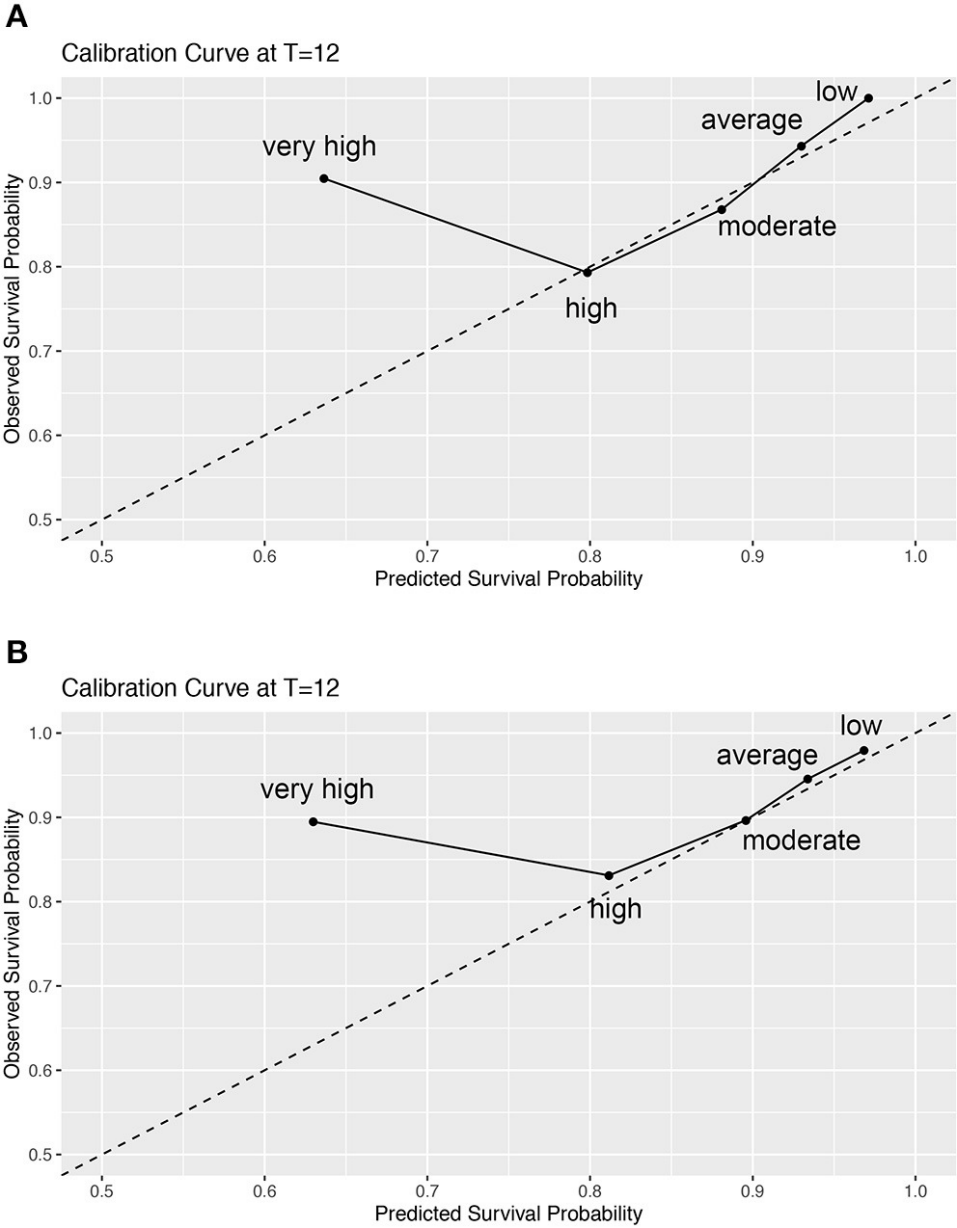
Calibration plots in the risk groups stratified by the REVEAL prognostic equation **(A)** and simplified risk calculator **(B)**. REVEAL, Registry to Evaluate Early and Long-term Pulmonary Arterial Hypertension Disease Management.

Additional analyses revealed satisfactory calibration in the low-risk, average-risk, moderately high-risk, and high-risk groups. In the very high-risk group, poor calibration resulted in a uniform overestimation of mortality risk (Figure 2). In other words, the observed risk was lower than the predicted risk. These results are consistent with the observed hazard ratios (Table 3), indicating that model discrimination and calibration were inaccurate for patients in the very high-risk groups.

## Discussion

The present study represents the first external validation of the REVEAL prognostic model in patients with SLE-PAH. Our findings suggest that the REVEAL prognostic equation exhibits reasonably good discrimination, and that such discrimination is better than that offered by the simplified risk calculator. Both prognostic models performed poorly in terms of calibration due to overestimation of risk in the highest risk group. Specifically, the REVEAL model may not perform well in predicting and separating 1-year OS in patients with SLE-PAH with very high-risk characteristics and the lowest predicted probabilities.

The REVEAL prognostic model has several strengths, including broad validation in large cohorts (5, 8, 20, 21), generalizability to patients with group 1 PAH, applicability to both prevalent and newly diagnosed cases, availability in the event of missing data, and rigorous derivation (6). However, the derivation cohort only enrolled 648 patients with CTD-PAH (23.9%), and most of them were SSC-PAH (13.5%). Due to the heterogeneity and complexity of CTD-PAH, the applicability of the REVEAL prognostic model in CTD-PAH requires further attention. A study involving patients with newly diagnosed SSC-PAH highlighted that REVEAL prognostic model should be interpreted with caution, given the potential for overestimation of risk in patients with low 6MWD scores of high serum BNP levels (10). Validation studies of the REVEAL tool for SSC-PAH are essential for determining whether the model can be useful in clinical practice (22). Although our results suggest that the REVEAL prognostic model exhibits reasonably good discriminative ability, the estimated risks may be unreliable because poor calibration can lead to misleading predictions (18). Differences in baseline characteristics and poorly fit variables may also have contributed to poor calibration.

Our validation cohort of patients with SLE-PAH exhibited a higher rate of pericardial effusion and included more young female adults than the REVEAL cohort of all patients with PAH. Although the proportion of patients with newly diagnosed PAH differed between the REVEAL and CSTAR-PAH cohorts, 1-year OS rates were similar. Differences between SLE-PAH and other types of PAH may explain these apparent discrepancies. In particular, our previous study demonstrated that PAH is significantly associated with serositis in patients with SLE (23), and that inflammation may be the major mechanism leading to serositis in patients with SLE-PAH (3). Such findings suggest that SLE-PAH is associated with a more inflammatory state than other forms of PAH. Previous studies have shown that patients with SLE-PAH were found to have a good clinic response to anti-inflammatory and immunosuppressive therapy (24, 25). Thus, strategies for optimal SLE control in patients with SLE-PAH are essential as part of the approach in the treatment of PAH.

The quality of the fit confirms the validity of the REVEAL model in patients with SLE-PAH. However, the model exhibited a marginal fit in patients with SLE-PAH. There are a few possible explanations for this finding, including earlier diagnosis of PAH in the validation cohort, more aggressive anti-inflammatory and immunosuppressive therapy in patients with SLE-PAH, differences in the distributions of missing values, insufficient sample size in the validation cohort, and clinical differences in disease progression. According to our results, renal insufficiency, WHO FC III, and heart rate >92 bpm contributed most to the poor model fit.

The risks of renal insufficiency and WHO FC III were higher in the CSTAR-PAH cohort than those in the REVEAL prognostic models. All patients with renal insufficiency at baseline in the CSTAR-PAH cohort had lupus nephritis and a relatively longer duration of SLE compare to the patients without renal insufficiency. In addition, lupus-related organ damage involving the renal system remains one of the major factors limiting survival improvement in patients with this disease (26). Indeed, patients with SLE exhibiting renal insufficiency have been shown to experience worse survival. In addition, patients in the SLE-PAH cohort were much younger than those in the REVEAL cohort. The reason for greater risk associated with WHO FC III in our cohort is uncertain. We speculate that the reporting of WHO FC III symptoms by young patients may be associated with greater disease severity than similar symptoms in older patients.

More notably, the risk assigned to a heart rate >92 bpm in the REVEAL prognostic models was not observed in our validation cohort. Sinus tachycardia is among the most commonly encountered heart rhythms that may portend an adverse prognosis, particularly in patients with cardiovascular disease (27). However, other factors may be responsible for sinus tachycardia in many patients with SLE, including inflammatory state, fever, anemia, pain, anxiety, or side effects related to corticosteroid use. These factors may have contributed to the poor model fit for patients with heart rates >92 bpm. Furthermore, only 6 patients had predicted diffusing capacity for carbon monoxide (DLCO) < 32% and three patients had mean right atrial pressure (mRAP) > 20 mmHg, while 8 patients had 6 MWD < 165 m. None of these patients died (Table 1). This is consistent with the weak predictive capability of single patient features and supports the need for multivariable risk stratification tools. Besides, the observational nature of this study could bias the results, given that patients with higher-risk disease may have been treated with more aggressive therapies.

Despite these inaccuracies, the REVEAL model may have powerful clinical applications in patients with SLE-PAH. Predicting survival is considered necessary for classifying patients with PAH due to rapid disease progression and high mortality rates. Since PAH is a multi-causal disease with diverse etiologies, no prediction model or risk stratification strategy will ever be able to predict all-cause deaths. Moreover, clinical decision-making may be more complex in some patients, necessitating more than a simple estimation of risk for all causes of death. However, quantification of risk enhances the shared decision-making process and may aid in the development of an effective decision-making tool. Therefore, updating the REVEAL model for application in patients with SLE-PAH may allow for more accurate prediction. To address this issue, a much larger sample size may be required.

The present study possesses several limitations of note. Very few patients in our validation cohort had 6MWD < 165 m (*n* = 8), DLCO <32% (*n* = 6), or mRAP > 20 mmHg (*n* = 3), so it was not possible to validate the true risk associated with these parameters (Table 1). The true risk of these predictors cannot be accurately estimated in our validation cohort. Further studies including larger cohorts of patients with SLE-PAH are required to assess the accuracy of these variables. In addition, patients with missing data were assigned to the reference group in accordance with the methods used for development of the REVEAL model. Therefore, missing data were unlikely to have contributed to the estimated risk. Either of these situations may have influenced model performance.

Our study is also limited in that the CSTAR-PAH cohort included more patients with newly diagnosed PAH than the REVEAL cohort. Indeed, more than half of patients in the CSTAR-PAH cohort were diagnosed within 1 year. Therefore, our study was only able to validate models for short-term prognosis in patients with SLE-PAH. Future studies should aim to evaluate the model's predictive value for longer-term outcomes, and to assess 1-year OS from any time point following RHC in patients with SLE-PAH. Given that some patients may exhibit a change in risk profile during follow-up, routinely re-assessments each year may be more appropriate.

Although, we validated the REVEAL tools, which were developed based on the largest registry of patients with PAH, there are other tools for risk assessment. Such tools include the ItinerAir-HTAP French Network on Pulmonary Hypertension registry (French registry) (28), pulmonary hypertension connection registry (PHC registry) (29), and Mayo Clinic PAH registry (Mayo registry) (30). Further research is required to validate the performance of these risk assessment tools in patients with SLE-PAH and compare them with the REVEAL model. In addition, the risk stratification strategy outlined in the 2015 European pulmonary hypertension guidelines (31) has been validated in several cohorts (32, 33). We were unable to calculate the risk probability and assess each risk factor for this strategy because the model was not as rigorously derived. Designed to facilitate use of the model in clinical practice, the updated REVEAL 2.0 risk score (34) can aid in determining PAH prognosis. However, without access to survival probabilities at the individual level, one cannot evaluate the calibration or fit of the model.

In summary, our findings indicate that the REVEAL prognostic equation exhibits reasonably good discriminative ability in patients with SLE-PAH, similar to findings observed in the REVEAL cohort. In contrast, the risk score calculator was associated with fair discrimination, which reflects poorer discriminative ability than in the REVEAL cohort. However, the model overestimated the risk of patients with SLE-PAH in the very high-risk group. Inaccurate estimates of renal insufficiency, WHO FC III, and heart rate >92 bpm contributed most to the poor model fit. Therefore, the results of our study should be considered when applying the REVEAL model for the prediction of 1-year OS in patients with SLE-PAH. Further studies are required to determine the most appropriate tools for predicting survival among patients with this distinct disease subtype.

## Data Availability Statement

The data analyzed during the current study is not publicly available since the data also forms part of an ongoing study, but is available from the corresponding author on reasonable request.

## Ethics Statement

The studies involving human participants were reviewed and approved by the Institutional Review Board of Peking Union Medical College Hospital (JS-2038). Written informed consent to participate in this study was provided by the participants' legal guardian/next of kin.

## Author Contributions

ML and XZeng is the guarantor and takes responsibility for the integrity of the work. All authors contributed to the acquisition and interpretation of the data. ML and JQu contributed to the conception and design of the study. JQu and YW performed the data analysis. JQu drafted the manuscript, and all authors critically revised the manuscript. All authors approved the final version for submission and agreed to be accountable for all aspects of the work.

## Conflict of Interest

The authors declare that the research was conducted in the absence of any commercial or financial relationships that could be construed as a potential conflict of interest.
